# Non-Invasive Pneumococcal Pneumonia in Portugal—Serotype Distribution and Antimicrobial Resistance

**DOI:** 10.1371/journal.pone.0103092

**Published:** 2014-07-30

**Authors:** Andreia N. Horácio, Joana P. Lopes, Mário Ramirez, José Melo-Cristino, for the Portuguese Group for the Study of Streptococcal Infections

**Affiliations:** Instituto de Microbiologia, Instituto de Medicina Molecular, Faculdade de Medicina, Universidade de Lisboa, Lisboa, Portugal; Instituto Butantan, Brazil

## Abstract

There is limited information on the serotypes causing non-invasive pneumococcal pneumonia (NIPP). Our aim was to characterize pneumococci causing NIPP in adults to determine recent changes in serotype prevalence, the potential coverage of pneumococcal vaccines and changes in antimicrobial resistance. Serotypes and antimicrobial susceptibility profiles of a sample of 1300 isolates recovered from adult patients (≥18 yrs) between 1999 and 2011 (13 years) were determined. Serotype 3 was the most frequent cause of NIPP accounting for 18% of the isolates. The other most common serotypes were 11A (7%), 19F (7%), 19A (5%), 14 (4%), 22F (4%), 23F (4%) and 9N (4%). Between 1999 and 2011, there were significant changes in the proportion of isolates expressing vaccine serotypes, with a steady decline of the serotypes included in the 7-valent conjugate vaccine from 31% (1999–2003) to 11% (2011) (P<0.001). Taking together the most recent study years (2009–2011), the potential coverage of the 13-valent conjugate vaccine was 44% and of the 23-valent polysaccharide vaccine was 66%. While erythromycin resistance increased from 8% in 1999–2003 to 18% in 2011 (P<0.001), no significant trend was identified for penicillin non-susceptibility, which had an average value of 18.5%. The serotype distribution found in this study for NIPP was very different from the one previously described for IPD, with only two serotypes in common to the ones responsible for half of each presentation in 2009–2011 – serotypes 3 and 19A. In spite of these differences, the overall prevalence of resistant isolates was similar in NIPP and in IPD.

## Introduction

Pneumonia is a common infection that causes high rates of morbidity and mortality worldwide. *Streptococcus pneumoniae* (pneumococcus) is thought to be the major cause of pneumonia, responsible for up to half of all cases [1]. Only a small fraction of pneumococcal pneumonias are bacteremic, with non-invasive pneumococcal pneumonia (NIPP) estimated to be three to ten-times more frequent than invasive pneumococcal pneumonia [2], [3]. While bacteremic pneumonia is a more severe form of pneumonia, it is less clear if bacteremia can be considered an independent predictor of mortality [4], [5]. In adults, bacteremic pneumonia accounts for most of the cases of invasive pneumococcal disease (IPD). While the serotype distribution of IPD and NIPP have been sometimes assumed to be the same [6], it is becoming increasingly clear that this is not so [5], [7]. This observation is in agreement with the recognition that some serotypes, and even different genetic lineages expressing the same serotype, may have different invasive disease potentials [8], leading to the expectation that less invasive serotypes would be more abundantly represented in NIPP than in IPD.

In developed countries, pneumonia is believed to be a major cause of morbidity among older adults, and, together with influenza, is the leading cause of death from infectious disease in the US considering the entire population [9]. Until recently, the only available vaccine for adults was the 23-valent polysaccharide vaccine (PPV23) that, while potentially effective in preventing IPD, may be less efficacious against NIPP [10], [11]. Possibly due to the ongoing debate on the usefulness of PPV23 vaccination, in the majority of the European countries, including Portugal, there has been a low uptake of this vaccine [12], [13]. On the other hand, the 7-valent conjugate vaccine (PCV7) was introduced in many European countries for vaccinating children, rapidly reaching high coverages in the targeted age groups. PCV7 was available in Portugal for vaccination of children between 2001 and 2009 and, although not part of the national immunization program, its uptake was estimated to have grown continuously, albeit slower that in countries where it was part of the national immunization program [14]. Changes in the serotype distribution of isolates causing IPD, compatible with an effect of PCV7 occurred in both children and adults in Portugal and elsewhere [13]–[17], the latter potentially resulting from a herd effect. The 10-valent (PCV10) and the 13-valent conjugate vaccines (PCV13) became available in Portugal in 2009 and in 2010, respectively. In September 2011, PCV13 received the European Medicines Agency approval for use in adults ≥50 yrs and in July 2013 was approved for adults ≥18 yrs, for the prevention of IPD. Currently PCV13 is approved for all ages from 6 weeks up and there is now initial evidence of its efficacy against pneumococcal pneumonia in adults caused by the serotypes included in the vaccine [18].

The aim of this study was to determine the serotype distribution and antimicrobial resistance of pneumococci causing NIPP in adults in Portugal during a 13-year period, when the three conjugate vaccines were available for children, and to compare these data to the information available for IPD published previously [19].

## Materials and Methods

### Ethics Statement

Case reporting and isolate collection were considered to be surveillance activities and were exempt from evaluation by the Review Board of the Faculdade de Medicina da Universidade de Lisboa. The data and isolates were de-identified so that these were irretrievably unlinked to an identifiable person.

### Bacterial Isolates

Isolates were provided by a laboratory-based surveillance system that includes 30 microbiology laboratories throughout Portugal. These were asked to identify and send to our laboratory all pneumococci causing infections. Although the laboratories were contacted periodically to submit the isolates to the central laboratory, no audit was performed to ensure compliance, which may be variable in this type of study. After arrival, all isolates were confirmed as *S. pneumoniae* by colony morphology and hemolysis on blood agar plates, optochin susceptibility and bile solubility. The isolates included in this study were recovered from adult patients (≥18 yrs) with a clinical diagnosis of pneumonia between 1999 and 2011. A total of 1300 isolates, 100 isolates randomly chosen from among the isolates received in each of the 13 years of the study were included. The total number of isolates submitted to the central laboratory in each year was 161 in 1999, 184 in 2000, 319 in 2001, 282 in 2002, 265 in 2003, 341 in 2004, 338 in 2005, 392 in 2006, 525 in 2007, 601 in 2008, 473 in 2009, 519 in 2010 and 445 in 2011. We believe this reflects increasing compliance with the surveillance activities with time. Only isolates recovered from sputum, bronchial secretions or bronchoalveolar lavage were considered. Isolates were not included when pneumococci were simultaneously isolated from blood or another usually sterile product, and when other potential bacterial pathogens besides pneumococci were detected in the sample (such as *Haemophilus influenzae* that was frequently detected). Only one isolate from each patient in each year was considered. Among the 1300 isolates selected, 103 (7.9%) were isolates from bronchoalveolar lavage fluid.

### Serotyping and Antimicrobial Susceptibility Testing

Serotyping was performed by the standard capsular reaction test using the chessboard system and specific sera (Statens Serum Institut, Copenhagen, Denmark). Serotypes were grouped into conjugate vaccine serotypes, i.e., those included in PCV13 (serotypes 1, 3, 4, 5, 6A, 6B, 7F, 9V, 14, 18C, 19F, 19A, 23F) that comprise all the serotypes found in the lower valency vaccines (PCV7: 4, 6B, 9V, 14, 18C, 19F, 23F; and PCV10: 1, 4, 5, 6B, 7F, 9V, 14, 18C, 19F, 23F), those included in PPV23 (all serotypes included in PCV13 except 6A and serotypes 2, 8, 9N, 10A, 11A, 12F, 15B, 17F, 20, 22F and 33F), and non-vaccine serotypes (NVT). The isolates that were not typable with any of the complete set of sera were considered non-typable (NT).

Minimum inhibitory concentrations (MICs) for penicillin and cefotaxime were determined using Etest strips (Biomérieux, Marcy-L'Etoile, France). The results were interpreted using the Clinical and Laboratory Standards Institute (CLSI) recommended breakpoints prior to 2008 [20], as these allows the comparison with previously published data. According to these criteria, intermediate level penicillin resistance is defined as MIC 0.12–1.0 µg/ml and high level resistance as MIC≥2.0 µg/ml. Isolates that fell into either one of these classes were designated penicillin non-susceptible. Susceptibility to cefotaxime was defined as MIC≤1.0 µg/ml. The Kirby-Bauer disk diffusion assay was used to determine susceptibility to levofloxacin, erythromycin, clindamycin, chloramphenicol, trimethoprim/sulphamethoxazole, tetracycline, vancomycin and linezolid, according to the CLSI recommendations and interpretative criteria [21]. Macrolide resistance phenotypes were identified using a double disc test with erythromycin and clindamycin, as previously described [22]. The MLS_B_ phenotype (resistance to macrolides, lincosamides and streptogramin B) was defined as the simultaneous resistance to erythromycin and clindamycin, while the M phenotype (resistance to macrolides) was defined as non-susceptibility only to erythromycin.

### Statistical Analysis

Sample diversity was measured using Simpson's index of diversity (SID) and the respective 95% confidence intervals (CI_95%_) [23]. To compare two sets of partitions the Adjusted Wallace (AW) coefficients were calculated [24] using the online tool available at www.comparingpartitions.info. Differences were evaluated by the Fisher exact test with the false discovery rate (FDR) correction for multiple testing [25] or the Chi-squared test, and the Cochran-Armitage test was used for trends. A P<0.05 was considered significant for all tests.

## Results

### Serotype Distribution

**Figure 1:**
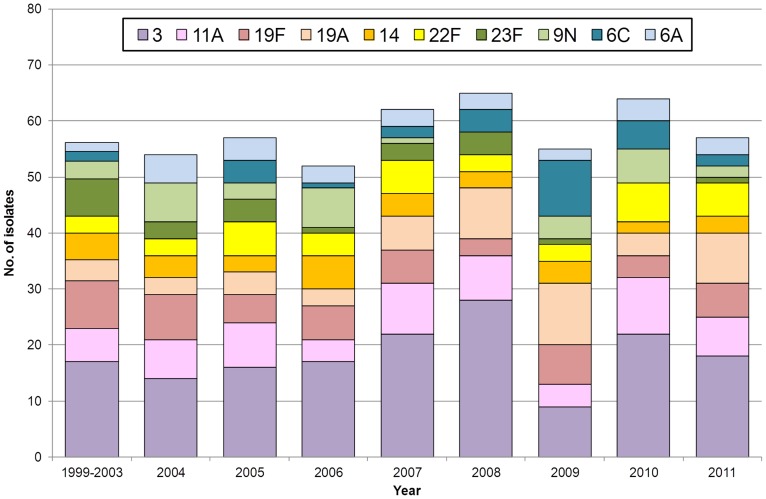
Serotype distribution of the isolates causing non-invasive pneumococcal pneumonia in adults in Portugal (1999–2011). Only the overall 10 most frequent serotypes are shown. The other serotypes found between 1999 and 2011 were serotypes 6B, 7F and 15A (n = 32, each), 23B (n = 30), 15B (n = 28), 10A (n = 27), 9V and non-typable (n = 26, each), 23A (n = 24), 1 (n = 22), 8 (n = 20), 16F (n = 19), 29 (n = 18), 4 (n = 17), 31 (n = 16), 34 (n = 15), 18C (n = 14), 17F and 33A (n = 13, each), 21 and 35F (n = 11, each), 15C and 35C (n = 10, each), 20 (n = 9), 12B and 17A (n = 8, each), 13, 25F and 25A/38 (n = 7, each), 7C and 28A (n = 5, each), 5 (n = 4), 11F, 18A and 19C (n = 3, each), 12A, 12F, 35A, 35B, 38 (n = 2, each) and 9L, 10F, 15F, 16A, 19B, 33F, 39 and 42 (n = 1, each). The value shown for 1999–2003 refers to the yearly average of the 500 isolates studied that were isolated in these 5 years. This period was analyzed together since previously published IPD data indicated that these corresponded to a pre-PCV7 serotype distribution [14].

Serotype diversity was high [SID: 0.941, CI_95%_: 0.935–0.948], with 57 different serotypes detected among the 1300 isolates. The most frequent serotypes, which accounted for more than half of the isolates, were serotypes: 3 (17.8%), 11A (6.7%), 19F (6.7%), 19A (5.2%), 14 (4.1%), 22F (4.1%), 23F (3.8%) and 9N (3.5%). Serotype distribution in each of the studied years is represented in Figure 1. We chose to represent an average of the yearly values between 1999 and 2003, because it was shown previously that this period corresponded to the years before an effect of children vaccination with PCV7 was noted in the distribution of adult IPD serotypes [14]. The yearly distribution on the 10 overall most frequent serotypes between 1999 and 2003 is represented in Figure S1. In spite of yearly variations, serotype diversity was high in all studied years with the lowest SID detected in 2008 [SID: 0.901, CI_95%_: 0.857–0.945] and the highest value found in both 2004 and 2009 [SID: 0.957, CI_95%_: 0.840–0.973].

**Figure 2:**
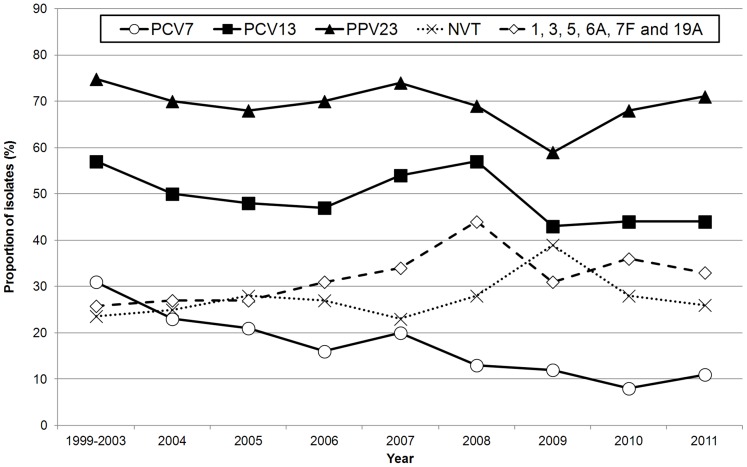
Proportion of isolates expressing serotypes included in pneumococcal vaccines causing non-invasive pneumococcal pneumonia in adults in Portugal (1999–2011). The value shown for 1999–2003 refers to the yearly average of the 500 isolates studied that were isolated in these 5 years. This period was analyzed together since previously published IPD data indicated that these corresponded to a pre-PCV7 serotype distribution [14].

The change in distribution of vaccine types along the study years is shown in Figure 2 and Figure S2. The serotypes included in PCV7 declined gradually from 31% in 1999–2003 to 11% in 2011 (Cochran-Armitage test of trend P<0.001). Among the PCV7 serotypes, those that contributed mostly to this decline were serotypes 6B (from 4.6% to 0%, Cochran-Armitage test of trend P<0.001), 9V (from 3.2% to 0%, Cochran-Armitage test of trend P<0.001) and 23F (from 6.6% to 1.0%, Cochran-Armitage test of trend P<0.001). Despite fluctuations throughout the study period in the number of isolates representing PCV13 and PPV23 serotypes, no consistent trend was noted. However, a decline of the isolates expressing serotypes included in these vaccines, and a consequent increase in the prevalence of NVTs, can be distinguished between 2008 and 2009. This can be attributed to a fall in serotype 3, from 28% to 9%, between these two years (P<0.001, Figure 1). Although in subsequent years the proportion of isolates expressing serotype 3 returned to values similar to those found previously (Figure 1), this did was not reflected in an increase in the proportion of isolates expressing PCV13 serotypes, which remained close to 43% (Figure 2). In contrast, the decline in the proportion of isolates expressing PPV23 serotypes noted in 2009 was not sustained, with the increases in the following years bringing this value back into the range found in the previous decade (Figure 2).

The distribution of the 10 most frequent serotypes found between 1999 and 2011, stratified by age group, is shown in table S1. The serotype distribution is similar for each of the age groups considered (P = 0.398) and no significant associations, after correction for FDR, could be found between specific serotypes and age groups. When considering only the three last years of the study, corresponding to the years immediately prior to PCV13 receiving approval for use in adults (2009–2011), the overall proportion of isolates expressing serotypes included in the various vaccines were 10.3% for PCV7, 43.7% for PCV13 and 66.0% for PPV23. There was also no correlation between the proportion of isolates causing NIPP included in the vaccines and the different age groups (table S2).

### Antimicrobial susceptibility

The proportion of isolates resistant to the tested antimicrobials between 1999 and 2011 is summarized in table 1. Penicillin non-susceptible pneumococci (PNSP) accounted for 18.5% of the isolates (n = 241). Among these, 211 isolates (16.2%) expressed low level resistance (MIC = 0.12–1 µg/mL) and 30 isolates (2.3%) high level resistance (MIC≥2 µg/mL). Considering the current CLSI guidelines for parenteral penicillin in non-meningitis cases, where high level resistance is defined as MIC≥8 µg/mL and intermediate resistance as MIC≥2 µg/mL [21], only 16 strains (1.2%) would have been considered non-susceptible to penicillin, with only one of these expressing high level resistance.

**Table 1:** Antimicrobial resistance of the isolates responsible for non-invasive pneumococcal pneumonia in adults in Portugal, stratified by age groups (1999–2011).

	No. resistant isolates (%)^a^			
	[18–49] yrs	[50–64] yrs	≥65 yrs	Total
	(n = 481)	(n = 293)	(n = 526)	(n = 1300)
PEN^b^	95 (19.8)	57 (19.5)	89 (16.9)	241 (18.5)
MIC90	0.25	0.38	0.25	-
MIC50	0.023	0.023	0.023	-
CTX	4 (0.8)	3 (1.0)	2 (0.4)	9 (0.7)
MIC90	0.19	0.125	0.094	-
MIC50	0.012	0.012	0.012	-
LEV	1 (0.2)	6 (2.0)	10 (1.9)	17 (1.3)
ERY	76 (15.8)	54 (18.4)	82 (15.6)	212 (16.3)
CLI	66 (13.7)	44 (15.0)	68 (12.9)	178 (13.7)
CHL	17 (3.5)	12 (4.1)	22 (4.2)	51 (3.9)
SXT	89 (18.9)	46 (15.7)	84 (16.0)	219 (16.8)
TET	61 (12.7)	42 (14.3)	63 (12.0)	166 (12.8)

a PEN – penicillin; CTX – cefotaxime; LEV – levofloxacin; ERY – erythromycin; CLI – clindamycin; CHL – chloramphenicol; SXT – trimethoprim/sulphamethoxazole; TET – tetracycline. All isolates were susceptible to vancomycin and linezolid.

b Non-susceptibitily to penicillin was determined using the CLSI breakpoints prior to 2008 [20].

Erythromycin resistant pneumococci (ERP) accounted for 16.3% of the isolates (n = 212), with 84.0% (n = 178) of these expressing the MLS_B_ phenotype and 16.0% (n = 34) the M phenotype. A total of 9.8% (n = 127) of the isolates were simultaneously non-susceptible to penicillin and resistant to erythromycin (EPNSP).

Resistance to levofloxacin was low overall (1.3%, n = 17), but higher in the older age groups than in the youngest group (Table 1, 18–49 yrs versus 50–64 yrs, P = 0.014 and 18–49 yrs versus ≥65 yrs, P = 0.012). No other significant associations with age were found for the other antimicrobials tested. All isolates were susceptible to vancomycin and linezolid.

The proportion of erythromycin and clindamycin resistant isolates increased between 1999–2003 and 2011. Erythromycin resistance increased from 8.0% to 18.0% (Cochran-Armitage test of trend P<0.001) and clindamycin resistance increased from 7.0% to 15.0% (Cochran-Armitage test of trend P = 0.004). No other significant changes were noted for the other antimicrobials tested.

There was an association between serotype and antimicrobial resistance. The AW for serotype and PNSP was 0.588 (CI_95%_: 0.541–0.634) and the AW for serotype and ERP was 0.489 (CI_95%_: 0.419–0.558). Table 2 shows the serotypes that presented at least 10 PNSP and ERP isolates, respectively. Among the major serotypes expressed by PNSP, only serotype 19F was not significantly associated with PNSP. Among the major serotypes expressed by ERP, only serotypes 23F and 6C were not significantly associated with ERP. The PCV7, PCV13 and PPV23 serotypes accounted for 56.0%, 70.5% and 69.7% of PNSP, respectively, and 42.9%, 60.8% and 66.5% of ERP, respectively.

**Table 2:** Serotype distribution of PNSP and ERP causing non-invasive pneumococcal pneumonia in adults in Portugal (1999–2011).

	Serotype^a^	No. of resistant isolates (%)	OR (CI_95%_)	P-value^b^
PEN	23F	39 (16.2)	12.6 (6.2–27.7)	**<0.001**
	14	37 (15.4)	8.1 (4.3–15.9)	**<0.001**
	19A	28 (11.6)	2.3 (1.3–3.9)	**0.002**
	15A	27 (11.2)	18.2 (6.8–61.3)	**<0.001**
	19F	27 (11.2)	1.4 (0.8–2.3)	0.193
	9V	23 (9.5)	25.5 (7.6–133.7)	**<0.001**
	6C	18 (7.5)	3.0 (1.5–6.2)	**0.001**
	Others^c^	42 (17.4)	-	-
ERY	19F	37 (17.5)	3.4 (2.1–5.4)	**<0.001**
	19A	31 (14.6)	3.7 (2.2–6.4)	**<0.001**
	15A	28 (13.2)	31.9 (11.0–126.2)	**<0.001**
	14	21 (9.9)	2.8 (1.5–5.1)	**<0.001**
	6B	15 (7.1)	3.7 (1.7–8.0)	**<0.001**
	23F	14 (6.6)	1.6 (0.8–3.1)	0.151
	33A	10 (4.7)	13.8 (3.5–79.0)	**<0.001**
	6C	10 (4.7)	1.5 (0.6–3.3)	0.296
	NT^d^	10 (4.7)	2.6 (1.0–6.1)	**0.025**
	Others^e^	36 (17.0)	-	-

a Only the serotypes that presented at least 10 non-susceptible isolates are shown.

b Significant P-values after FDR correction are highlighted in bold.

c Other serotypes found among PNSP: 6B (n = 8), non-typable (n = 7), 6A and 29 (n = 5, each), 23B and 24F (n = 3, each), 7C (n = 2), 1, 3, 4, 11A, 15B, 15F, 22F, 23A, 35A (n = 1, each).

d NT – non typable.

e Other serotypes found among ERP: 9V and 11A (n = 4, each), 3, 15B, 22F, 23A, 24F (n = 3, each), 6A (n = 2), 1, 7F, 8, 9N, 15F, 16F, 17F, 23B, 29, 33F and 35F (n = 1, each).

## Discussion

Serotype 3 was the most important serotype in NIPP in adults in Portugal. This serotype was the most frequently detected in all studied years, with the exception of 2009, when it ranked third (Figure 1). A predominance of serotype 3 was also found in two studies that focused on the serotype distribution of pneumococcal pneumonia isolates [5], [26], and this serotype was among the most frequent in two recent studies using urinary antigen detection assays to diagnose pneumococcal pneumonia [7], [27], although these last studies included both NIPP and bacteremic pneumonia. In Portugal, serotype 3 is not only a leading cause of NIPP but also of IPD, as can be seen in Figure S3, showing the distribution of the most frequently detected serotypes immediately prior to the licensure of PCV13 for adult immunization (2009–2011).

**Figure 3:**
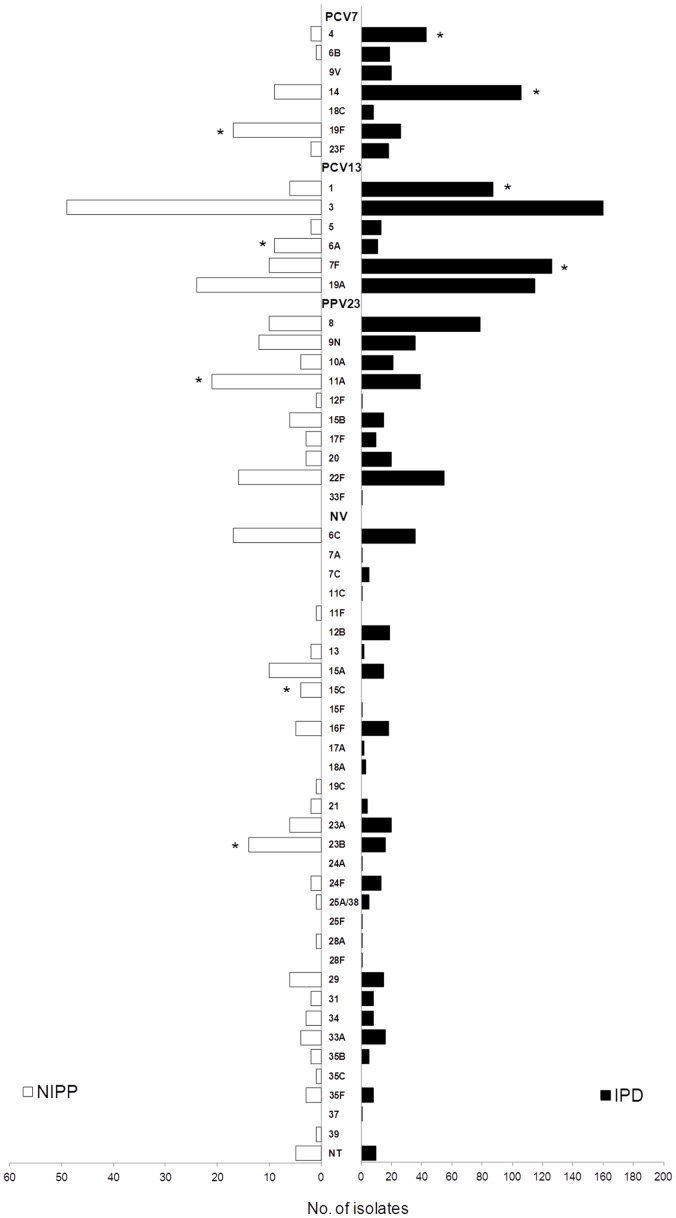
Serotype distribution of the isolates causing non-invasive pneumococcal pneumonia and invasive pneumococcal disease in adults in Portugal (2009–2011). Data from IPD were published previously [19]. Serotypes associated with NIPP or IPD are marked by asterisks. The odds ratio was used to measure the association between serotype and disease presentation and only significant values (P<0.05) after FDR correction are indicated. The P values for the serotypes associated with NIPP were: P = 0.001 for 19F, P = 0.007 for 6A, P = 0.004 for 11A, P = 0.001 for 15C, P<0.001 for 23B. The P values for the serotypes associated with IPD were: P = 0.007 for 4, P<0.001 for 14, P<0.001 for 1 and P<0.001 for 7F.

Although both IPD and NIPP were characterized by a high serotype diversity (for NIPP SID = 0.943, CI_95%_: 0.932–0.955; and for IPD SID = 0.942, CI_95%_: 0.937–0.946; considering 2009–2011), the actual serotype distribution is quite different (Figures 3, Figure S3 and Table S3). Among the most frequent serotypes, accounting for half of the characterized isolates in 2009–2011, only two serotypes were common to both NIPP and IPD, which were serotypes 3 and 19A (Figure S3). When comparing the serotype distribution, serotypes 6A, 11A, 15C, 19F and 23B were significantly more abundant among NIPP isolates, whereas serotypes 1, 4,7F and 14 were significantly more abundant among IPD isolates (Figure 3 and Table S3). These four serotypes, together with serotype 3, were already shown to have an enhanced invasive disease potential in a study evaluating the serotypes and clones circulating in Portugal [8]. On the other hand, serotypes 6A, 11A and 19F were associated with carriage [8], suggesting their lower invasive disease potential, consistent with the association with NIPP determined here (Figure 3 and Table S3).

A recent study described the serotype distribution among isolates recovered in 2011 causing bacteremic and non-bacteremic pneumonia in adults in Denmark [5]. When considering only the isolates recovered in 2009–2011 in Portugal, the serotype distribution is, perhaps surprisingly, remarkably similar in both studies. Among the differences are the more significant fractions of non-typable isolates among NIPP and of serotype 1 isolates in bacteremic pneumonia in Denmark relative to Portugal. Serotype 1 was always an important serotype among isolates causing IPD in both children and adults in Portugal, but its significance has declined in recent years [13], [15], [19], [28]. This change cannot be attributed entirely to the use of higher valency vaccines, such as PCV13, although vaccination was potentially a contributing factor [19], [28]. Another important difference is the persistence of serotype 14 isolates among both NIPP and IPD in Portugal, in contrast to Denmark where this serotype was found at a lower frequency [5]. Serotype 14 was already included in PCV7, and continued vaccine use could be expected to significantly reduce its prevalence. A lower and more protracted vaccine uptake in Portugal compared to Denmark, together with a higher antibiotic consumption, could contribute to the differences observed between the two countries.

In previous studies, we showed that immunization of children with PCV7 resulted in the decline of PCV7 serotypes as causes of adult IPD [13], [19]. In the present study, we show that a decline of PCV7 serotypes also occurred among isolates causing NIPP in adults. However, while in IPD this decline was abrupt, occurring between 2004 and 2005, in NIPP this decline was gradual and occurred over the entire post-PCV7 period (Figure 2 and Figure S2). The proportion of isolates expressing PCV7 serotypes in the most recent years of the study was different between NIPP and IPD (10.3% versus 19.0% in 2009–2011, P<0.001, Figure 3).

Taken together, the isolates expressing PCV13 serotypes also declined in IPD and in NIPP, a change that was observed from 2008 onwards for both disease presentations. However, again there were important differences. While in IPD this decline occurred from 2008 to 2011 and was mostly due to decreases in serotypes 1 and 5, in NIPP this decline occurred between 2008 and 2009 and was predominantly caused by a decrease in serotype 3. In neither case can we attribute these changes to the introduction of PCV13 in Portugal, since this vaccine only became available for children in the beginning of 2010 and received an indication for adults in the beginning of 2012. In NIPP, one possible explanation for the change observed could be the H1N1pdm09 pandemic that occurred between 2009 and 2010. Individuals infected by influenza are at high risk of developing secondary bacterial infections, especially with pneumococci [29]. Consistent with the hypothesis that influenza allowed the emergence of multiple serotypes as causes of NIPP, the decrease of serotype 3 from 2008 to 2009 was accompanied by an increase in serotype diversity.

Another remarkable difference between NIPP and IPD is the proportion of isolates that expresses serotypes included in the available vaccine formulations with an adult indication. When analyzing data from 2009 to 2011, we found that the number of IPD cases that could have been potentially prevented by PCV13 and PPV23 was 59%, and 80%, respectively [19], while the proportion of isolates expressing these serotypes was only 44% and 66%, respectively, among our collection of NIPP isolates. The higher proportion of vaccine types among IPD isolates was also documented in Denmark [5]. The efficacy of the conjugate vaccines is well established and adults could now benefit directly from PCV13 use. However, according to our sample more than half of NIPP cases could not have been prevented by vaccination with PCV13.

In the present study we could not find any significant associations between serotypes and age groups. This is in contrast to our previous studies with invasive isolates, where serotypes 3 and 19A were associated with older patients and serotypes 1 and 8 were associated with younger patients [19]. However, if we do not consider the correction for multiple testing, serotype 3 was more frequent in older adults than in the youngest (14% in 18–49 yrs versus 20% in ≥50 yrs, P = 0.005, Table S1). The lack of association for the other three serotypes with age is likely the result of their small numbers in our NIPP sample, particularly in what concerns serotypes 1 and 8.

A high proportion of the resistant isolates recovered between 1999 and 2011 are of serotypes included in PCV7 (Table 2), accounting for 56% of PNSP and 43% of ERP in the entire study period. Unlike what could have been expected, the introduction of PCV7 in Portugal did not reduce the proportion of resistant isolates, neither in NIPP nor in IPD [14], [19]. Actually, for both presentations there was an increase in ERP between 1999 and 2011, and for IPD there was also an increase in PNSP. However, when we considered the most recent data (2009 to 2011) we found that only 22% of PNSP and 26% of ERP causing NIPP, represented serotypes included in PCV7. This means that resistant isolates expressing serotypes that are not included in PCV7 have emerged and expanded in the post-PCV7 period.

When considering the entire study period, antimicrobial resistance among NIPP isolates was similar to the values reported recently for IPD [19] (Table 1). Given the association between serotype and antimicrobial resistance, and the different serotype distributions between NIPP and IPD, how can we explain the similar overall resistance? For the most part, the explanation can be found in the more gradual decrease of resistant PCV7 serotypes, albeit to a lower level, in NIPP when compared to IPD. This was accompanied by the rise of a different set of serotypes including resistant isolates that are not included in PCV7, resulting in similar overall resistance levels. Resistance among NIPP isolates is partly due to the proliferation of resistant serotype 19A isolates, probably representing a lineage which has been expanding as a cause of IPD in children and adults [19], [30], and that became the single most important serotype among PNSP and ERP in the last three years of the study. This was accompanied by increases in serotypes including resistant isolates not represented in PCV13, such as serotypes 6C, 15A, 29, 33A, as well as non-typable isolates, each including n>5 PNSP or ERP during the entire study period (table 2).

The major limitation of this study is that we do not know if blood cultures were performed for all patients, and so we cannot exclude the possibility that some of the isolates attributed to NIPP were in fact reflecting cases of invasive disease. However, the distinct serotype distribution between IPD and NIPP and the similar distribution found in this study and among isolates causing NIPP in Denmark in a similar period [5], in spite of the different epidemiological contexts, strongly argues against a significant bias in our sample. Another possible confounder could be that a fraction of our isolates are reflecting colonization and not infection. Again we consider this unlikely. The fluids included are not present in healthy subjects (sputum and bronchial secretions) or are not obtained unless there is a strong suspicion of pneumonia (bronchoalveolar lavage). The participating laboratories used criteria to exclude low quality samples, which would be more likely to reflect upper airway microbiota. Finally, adult colonization is known to be rare [31] and would be therefore unlikely to account for a significant fraction of the isolates. Taken together, these arguments support a role for the pneumococci analyzed in infection and not asymptomatic colonization. The decision to collect specimens for microbiological analysis was the responsibility of the attending physician that did not receive specific guidelines. We are not aware of significant changes in practice during the study period, although differences between the participating centers may exist. However, since these are expected to be minor and stable during the study period we do not feel these constitute a significant source of bias.

In this study, we found a different serotype distribution and dynamics in NIPP and IPD in the same population. This was highlighted by the fact that the potential coverage of the currently available pneumococcal vaccines with an adult indication is lower in NIPP than in IPD. The distinct dynamics of NIPP, the availability of PCV13 for adults together with the issues raised regarding the efficacy of PPV23 in the context of NIPP, and the fact that NIPP is a frequent cause of morbidity and mortality among adults, all underscore the relevance of considering the use of PCV13 in adults. However, the expected herd protection conferred by vaccinating children with PCV13 could reduce the benefits of direct adult vaccination. We documented here ongoing changes in the serotypes causing NIPP that are potentially due to long-term PCV7 use, but there is uncertainty regarding the ultimate reduction in vaccine serotypes one can expect from this effect, as well as regarding the kinetics of such a decline. Continued surveillance is essential to evaluate the changing potential benefits of direct adult vaccination.

## Supporting Information

Figure S1 **Proportion of isolates of each of the serotypes that together were responsible for half of non-invasive pneumococcal pneumonia isolates and half of invasive pneumococcal disease cases in adults in Portugal (2009–2011).** Data from IPD were published previously [19].
(PDF)

Figure S2 **Proportion of isolates expressing serotypes included in pneumococcal vaccines causing non-invasive pneumococcal pneumonia in adults in Portugal (1999–2003).**
(PDF)

Figure S3 **Proportion of isolates of each of the serotypes that together were responsible for half of non-invasive pneumococcal pneumonia isolates and half of invasive pneumococcal disease cases in adults in Portugal (2009–2011).** Data from IPD were published previously (19).
(PDF)

Table S1 **Serotype distribution of the 10 most common serotypes responsible for non-invasive pneumococcal pneumonia in adults in Portugal, stratified by age groups (1999–2011).**
(PDF)

Table S2 **Isolates expressing vaccine serotypes responsible for non-invasive pneumococcal pneumonia in adults in Portugal, stratified by age groups (2009–2011).**
(PDF)

Table S3 **Serotype distribution of the 10 overall most common serotypes in NIPP and in IPD (2009–2011).**
(PDF)
