# Supplementary material for: Non-Invasive Pneumococcal Pneumonia in Portugal—Serotype Distribution and Antimicrobial Resistance
Source: PLoS One. 2014 Jul 30;9(7):e103092. doi: 10.1371/journal.pone.0103092 (PMC4116175; doi:10.1371/journal.pone.0103092)
Supplement: Figure S1 — Proportion of isolates of each of the serotypes that together were responsible for half of non-invasive pneumococcal pneumonia isolates and half of invasive pneumococcal disease cases in adults in Portugal (2009–2011). Data from IPD were published previously [19]. (PDF) [file pone.0103092.s001.pdf]

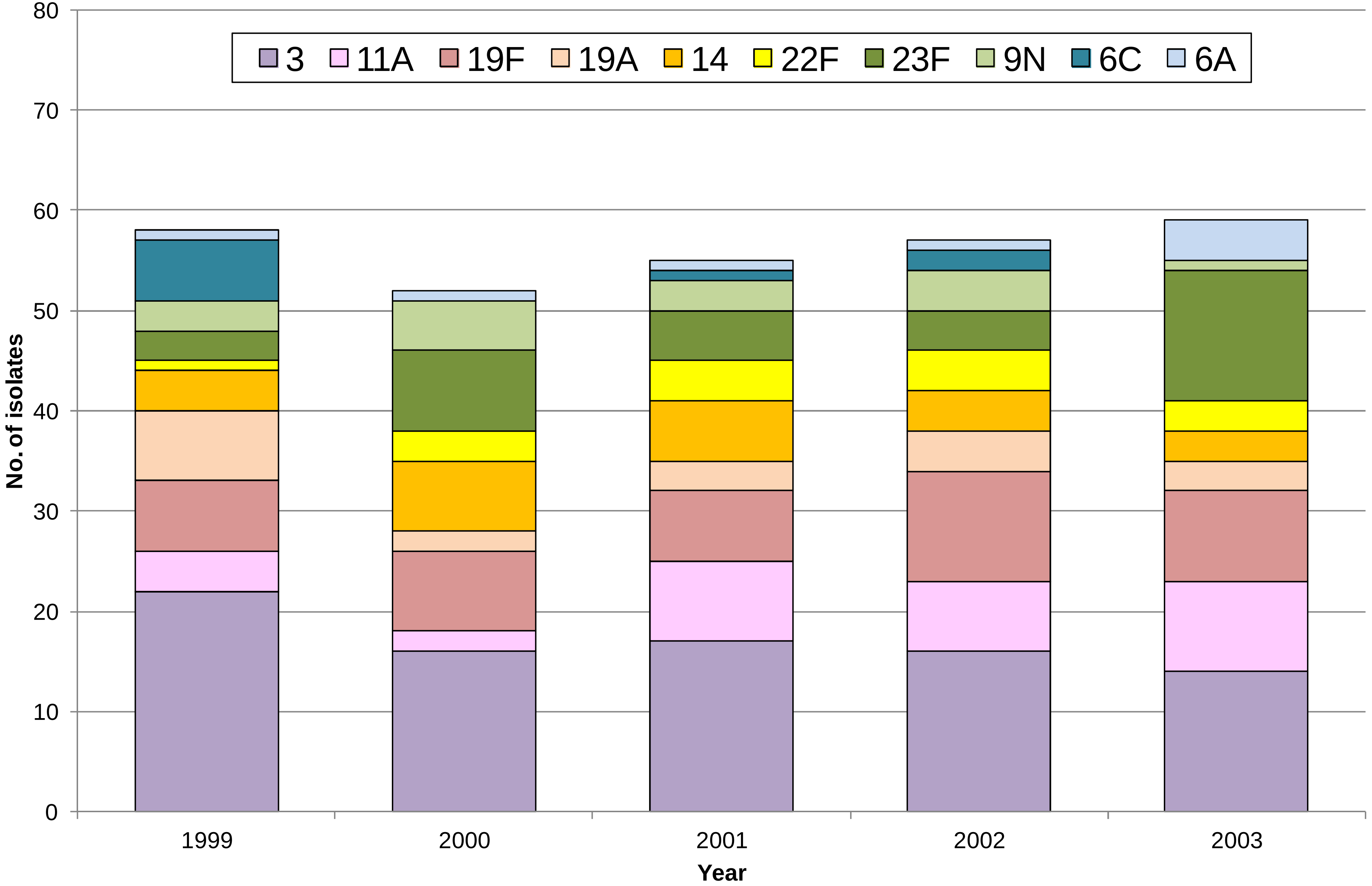

Figure S1 – Serotype distribution of the isolates causing non-invasive pneumococcal pneumonia in adults in Portugal (1999-2003). Only the overall 10 most frequent serotypes are shown.
