# Supplementary material for: Non-Invasive Pneumococcal Pneumonia in Portugal—Serotype Distribution and Antimicrobial Resistance
Source: PLoS One. 2014 Jul 30;9(7):e103092. doi: 10.1371/journal.pone.0103092 (PMC4116175; doi:10.1371/journal.pone.0103092)
Supplement: Figure S2 — Proportion of isolates expressing serotypes included in pneumococcal vaccines causing non-invasive pneumococcal pneumonia in adults in Portugal (1999–2003). (PDF) [file pone.0103092.s002.pdf]

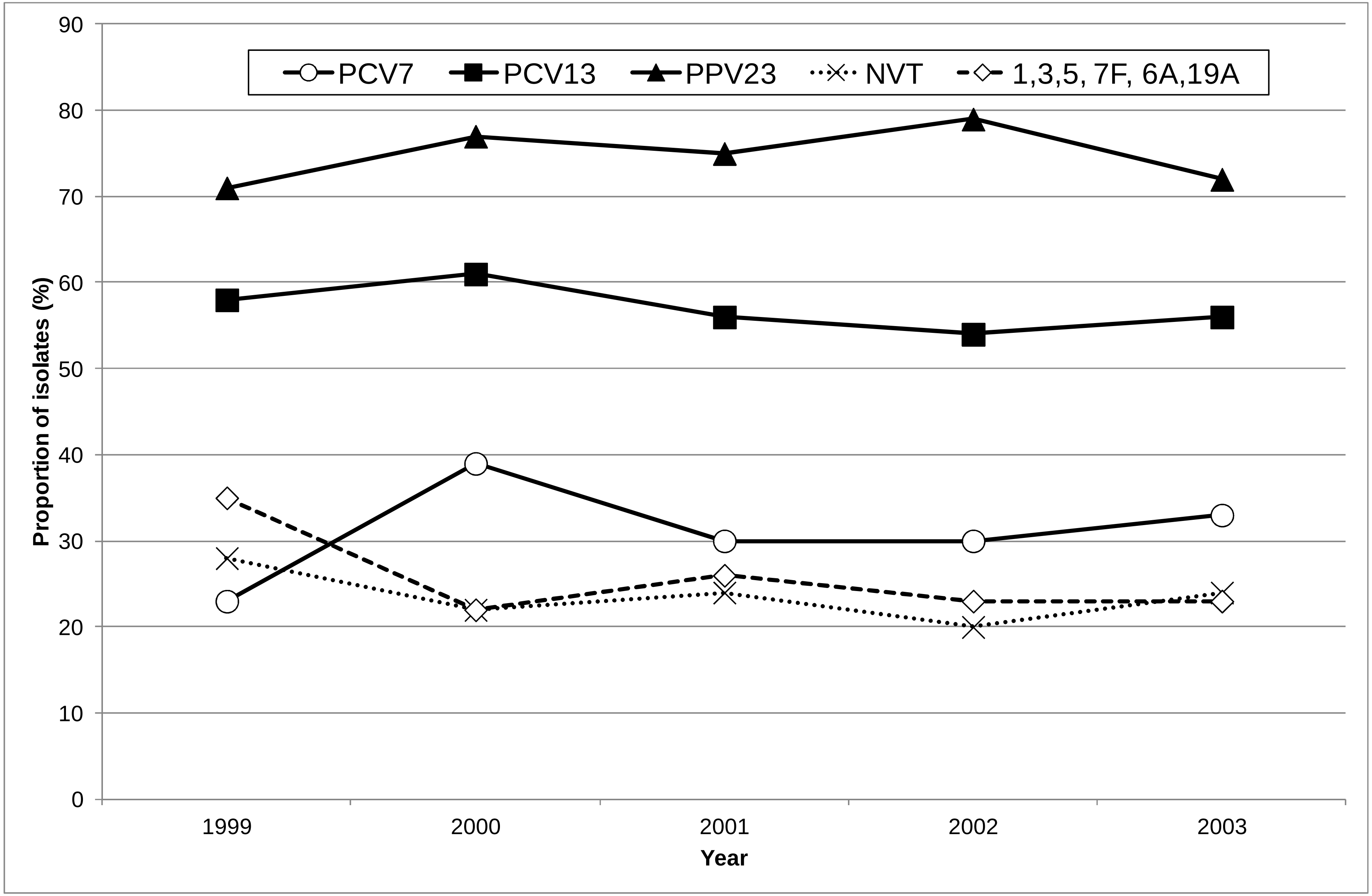

Figure S2 – Proportion of isolates expressing serotypes included in pneumococcal vaccines causing non-invasive pneumococcal pneumonia in adults in Portugal (1999-2003).
