# Supplementary material for: Non-Invasive Pneumococcal Pneumonia in Portugal—Serotype Distribution and Antimicrobial Resistance
Source: PLoS One. 2014 Jul 30;9(7):e103092. doi: 10.1371/journal.pone.0103092 (PMC4116175; doi:10.1371/journal.pone.0103092)
Supplement: Figure S3 — Proportion of isolates of each of the serotypes that together were responsible for half of non-invasive pneumococcal pneumonia isolates and half of invasive pneumococcal disease cases in adults in Portugal (2009–2011). Data from IPD were published previously (19). (PDF) [file pone.0103092.s003.pdf]

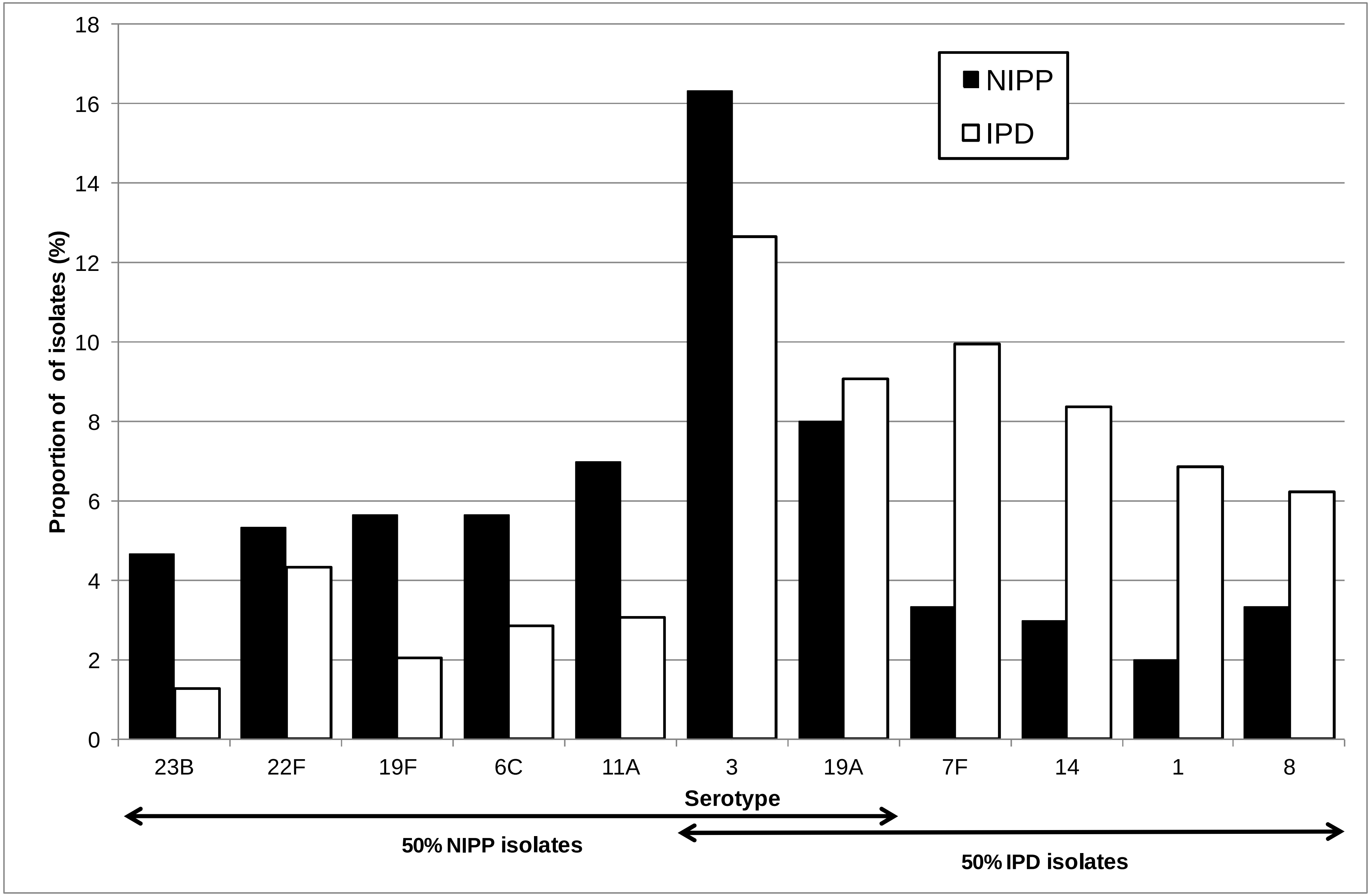

Figure S3 – Proportion of isolates of each of the serotypes that together were responsible for half of non-invasive pneumococcal pneumonia isolates and half of invasive pneumococcal disease cases in adults in Portugal (2009-2011). Data from IPD were published previously (19).
