# Supplementary material for: Non-Invasive Pneumococcal Pneumonia in Portugal—Serotype Distribution and Antimicrobial Resistance
Source: PLoS One. 2014 Jul 30;9(7):e103092. doi: 10.1371/journal.pone.0103092 (PMC4116175; doi:10.1371/journal.pone.0103092)
Supplement: Table S1 — Serotype distribution of the 10 most common serotypes responsible for non-invasive pneumococcal pneumonia in adults in Portugal, stratified by age groups (1999–2011). (PDF) [file pone.0103092.s004.pdf]

Table S1: Serotype distribution of the 10 most common serotypes responsible for non-invasive pneumococcal pneumonia in adults in Portugal, stratified by age groups (1999-2011)

	No. isolates (%)		
	[18-49] yrs (n=481)	[50-64] yrs (n=293)	≥65 yrs (n=526)
3	67 (13.9)	63 (21.5)	101 (19.2)
11A	29 (6.0)	16 (5.5)	42 (8.0)
19F	36 (7.5)	16 (5.5)	35 (6.7)
19A	25 (5.2)	15 (5.1)	28 (5.3)
14	20 (4.2)	13 (4.4)	20 (3.8)
22F	18 (3.7)	9 (3.1)	26 (4.9)
23F	17 (3.5)	10 (3.4)	23 (4.4)
9N	21 (4.4)	6 (2.0)	19 (3.6)
6C	16 (3.3)	8 (2.7)	13 (2.5)
6A	10 (2.1)	10 (3.4)	15 (2.9)
Other	222 (46.2)	127 (43.3)	204 (38.8)
