# Supplementary material for: Non-Invasive Pneumococcal Pneumonia in Portugal—Serotype Distribution and Antimicrobial Resistance
Source: PLoS One. 2014 Jul 30;9(7):e103092. doi: 10.1371/journal.pone.0103092 (PMC4116175; doi:10.1371/journal.pone.0103092)
Supplement: Table S2 — Isolates expressing vaccine serotypes responsible for non-invasive pneumococcal pneumonia in adults in Portugal, stratified by age groups (2009–2011). (PDF) [file pone.0103092.s005.pdf]

Table S2. Isolates expressing vaccine serotypes responsible for non-invasive pneumococcal pneumonia in adults in Portugal, stratified by age groups (2009-2011)

	No. isolates (%)		
	[18-49] yrs	[50-64] yrs	≥65 yrs
PCV7	9 (12.0)	10 (12.2)	12 (8.4)
PCV13	34 (45.3)	39 (47.6)	58 (40.6)
PPV23	45 (60.0)	57 (69.5)	96 (67.1)
