# Supplementary material for: Non-Invasive Pneumococcal Pneumonia in Portugal—Serotype Distribution and Antimicrobial Resistance
Source: PLoS One. 2014 Jul 30;9(7):e103092. doi: 10.1371/journal.pone.0103092 (PMC4116175; doi:10.1371/journal.pone.0103092)
Supplement: Table S3 — Serotype distribution of the 10 overall most common serotypes in NIPP and in IPD (2009–2011). (PDF) [file pone.0103092.s006.pdf]

Table S3. Serotype distribution of the 10 overall most common serotypes in NIPP and in IPD (2009-2011).

NIPP			IPD*		
Serotype†	n (%)	Cumulative n (%)	Serotype†	n (%)	Cumulative n (%)
3	49 (16.3)	49 (16.3)	3	160 (12.6)	160 (12.6)
19A	24 (8.0)	73 (24.3)	**7F**	126 (10.0)	286 (22.6)
**11A**	21 (7.0)	94 (31.3)	19A	115 (9.1)	401 (31.7)
6C	17 (5.7)	111 (37.0)	**14**	106 (8.4)	507 (40.1)
19F	17 (5.7)	128 (42.7)	1	87 (6.9)	594 (47.0)
22F	16 (5.3)	144 (48.0)	8	79 (6.2)	673 (53.2)
**23B**	14 (4.7)	158 (52.7)	22F	55 (4.3)	728 (57.5)
9N	12 (4.0)	170 (56.7)	**4**	43 (3.4)	771 (60.9)
7F	10 (3.3)	180 (60.0)	11A	39 (3.1)	810 (64.0)
8	10 (3.3)	190 (63.3)	6C	36 (2.8)	846 (66.0)

* Information published previously (19)

†Serotypes in bold were associated with NIPP or IPD (P<0.05)
